# Partial Horner’s Syndrome Following Thyroidectomy Without Lateral Neck Dissection: A Rare Case Report and Literature Review

**DOI:** 10.1155/crie/1253094

**Published:** 2026-04-23

**Authors:** Eve Hopping, Femi E. Ayeni, Senarath Edirimanne

**Affiliations:** ^1^ Dept of Surgery, Nepean Hospital, Penrith, New South Wales, Australia, nbmlhd.health.nsw.gov.au; ^2^ Faculty of Medicine and Health, The University of Sydney, Sydney, New South Wales, Australia, sydney.edu.au; ^3^ Nepean Institute of Academic Surgery, Nepean Clinical School, The University of Sydney, Penrith, New South Wales, Australia, sydney.edu.au

**Keywords:** cervical sympathetic chain, Horner’s syndrome, neck dissection, thyroidectomy

## Abstract

Horner’s syndrome is a rare complication of thyroid surgery and is most commonly reported as a complication associated with lateral neck dissection. We present the case of a 46‐year‐old woman who developed a partial Horner’s syndrome following hemithyroidectomy and central neck dissection, without lateral neck dissection. The patient underwent right hemithyroidectomy and central neck dissection for a TIRADS‐5 nodule and developed right sided facial anhidrosis and absence of right‐sided facial erythema on exertion 6 weeks post‐operatively. No ocular involvement was detected. Neurologist review confirmed partial Horner’s syndrome, which eventually resolved. The literature regarding development of Horner’s syndrome as a complication of thyroid surgery without lateral neck dissection was reviewed via PubMed. All English language publications of adult patients developing features of Horner’s syndrome after open thyroid surgery (hemithyroidectomy or thyroidectomy, +/− central neck dissection) were included. Twenty one cases of Horner’s syndrome following thyroid surgery without lateral neck dissection were identified. Interestingly, only four of these cases featured anhidrosis, and no case reported asymmetrical facial erythema. Four possible mechanisms of cervical sympathetic chain dysfunction resulting in Horner’s syndrome post‐thyroidectomy have been proposed: neuropraxia from retraction; mass effect from post‐operative haematoma; ischaemia‐induced nerve damage; damage to communicating fibres during repeated inspection of the recurrent laryngeal nerve. To our knowledge, this is the only reported case of a post‐thyroidectomy partial Horner’s syndrome with associated asymmetrical flushing.

## 1. Introduction

Horner’s syndrome is a pattern of symptoms and signs caused by disruption of the oculosympathetic neural pathway. The classical presentation of Horner syndrome is ptosis, pupillary miosis and facial anhidrosis. Horner’s syndrome was first described in 1852 by French physiologist Claude Bernard, and then, in 1869 by Swiss ophthalmologist Johann Friedrich Horner [1].

In a complete Horner’s syndrome, disruption of sympathetic innervation to the pupillary dilator muscle results in a loss of opposition to the parasympathetically innervated pupillary constrictor and resultant pupillary constriction (miosis). Disruption of sympathetic innervation to the superior tarsal muscle (aka Mueller’s muscle) of the upper eyelid results in subtle ptosis [2]. Loss of sympathetic innervation to sweat glands of the ipsilateral face and neck results in anhidrosis [3]. Less commonly, disruption to sympathetic innervation to the skin through vasodilator neurones results in ipsilateral absence of flushing on exertion, also referred to as the harlequin sign [4, 5].

A Horner’s syndrome arises due to disruption of the oculosympathetic neural pathway anywhere along its’ course. This pathway begins at the hypothalamus, proceeds to the brainstem and spinal cord and synapses in C8‐T2 of the spinal cord. Second order neurones then ascend through the brachial plexus over the apex of the lung and through the neck as the sympathetic chain to synapse within the superior cervical ganglion between the level of C2‐C4. Third order neurones exit the superior cervical ganglion and innervate their target organs (the eye as the long and short ciliary nerves; the sweat glands as sympathetic fibres travelling with the external carotid) [2].

In the context of thyroid surgery, the cervical sympathetic chain is the part of the oculosympathetic pathway in closest proximity to the operative field. The sympathetic chain lies deep to the prevertebral facia. In conventional anatomy, the cervical sympathetic trunk runs posterior or postero‐medial to the carotid sheath; however, it has also been reported to be found within the sheath [3]. In conventional anatomy, the sympathetic trunk contains four ganglia: the superior cervical, middle cervical, inferior cervical aka cervical thoracic and vertebral ganglia.

**Figure 1:**
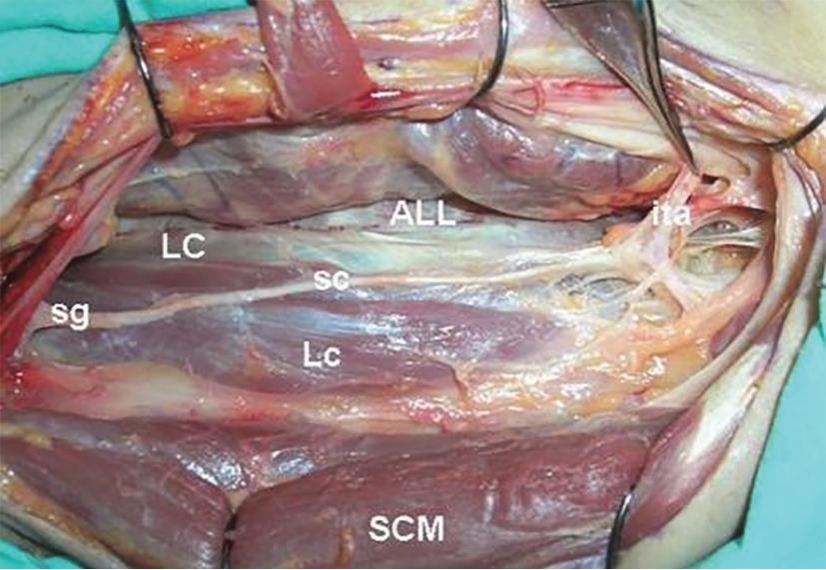
Relationship between sympathetic chain and inferior thyroid artery. Dissection highlighting the close association between sympathetic trunk (sc) and inferior thyroid artery (ita). ALL, anterior longitudinal ligament; Lc, longus capitis; LC, longus colli; SCM, sternocleidomastoid; sg, superior ganglion of sympathetic trunk.

**Figure 2:**
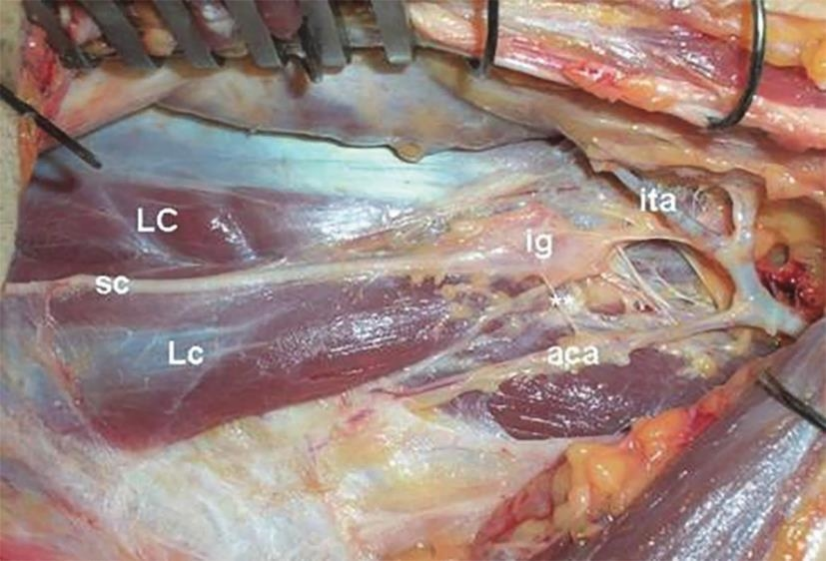
Relationship between intermediate cervical ganglion and inferior thyroid artery. Reproduced with permission from Civelek et al [6]. aca, ascending cervical artery; ig, intermediate (middle) ganglion of sympathetic trunk; ita, inferior thyroid artery; Lc, longus capitis; LC, longus colli; sc, sympathetic trunk. Double asterisks, lateral attachment of sympathetic trunk (grey rami communicantes).

The superior cervical ganglion is the largest of the four and is conventionally located posterior to the internal carotid artery at the level of C2–C4 [6]. The middle cervical ganglion lies at the level of C6, in proximity to the inferior thyroid artery [6, 7]. While cadaveric studies suggest that the location of the superior and middle cervical ganglia are relatively consistent [6], reported variations abound, including the potential for the inferior thyroid artery to cross the middle cervical ganglion either anteriorly or posteriorly (Figures 1 and 2), giving rise to the potential for nerve injury during ligation of the artery. Horner’s syndrome is a rare complication of thyroid surgery, with an incidence previously reported at 0.2% [8, 9].

However, most cases in the literature describe a Horner’s syndrome following thyroid surgery with associated lateral neck dissection. Horner’s syndrome is a logical complication of lateral neck dissection, as this places the conventional location of the cervical sympathetic chain is within the operative field. However, thyroidectomy with dissection limited to the central neck compartment alone should not cross the path of cervical sympathetic chain, making it more challenging to understand the mechanism of this complication in cases without lateral neck dissection.

The goal of this manuscript is to examine the cases of Horner’s syndrome following thyroidectomy without lateral central neck dissection and to review potential mechanisms and aetiologies of this complication, and thus, help to prevent future occurrences.

## 2. Case Presentation

We present the case of a 46 year old woman who initially presented with a right‐sided neck lump. Past medical history was significant for resection of a meningioma resulting in right sided tinnitus, visual impairment and ptosis; duodenal neuroendocrine tumour under surveillance; previous cutaneous BCC excisions; cholecystectomy; oesophageal mucosal resection. There was no history of prior neck surgery or radiation.

Ultrasound revealed a 13 mm TIRADS‐5 right mid‐pole thyroid nodule., Cytology was reported as Bethesda Category 5. The patient proceeded for right hemithyroidectomy and central neck dissection. The intraoperative course was unremarkable, and neuromonitoring was utilised throughout. Histology confirmed classical papillary thyroid cancer, BRAF positive. 1/5 nodes from central neck dissection were positive for metastasis.

**Figure 3:**
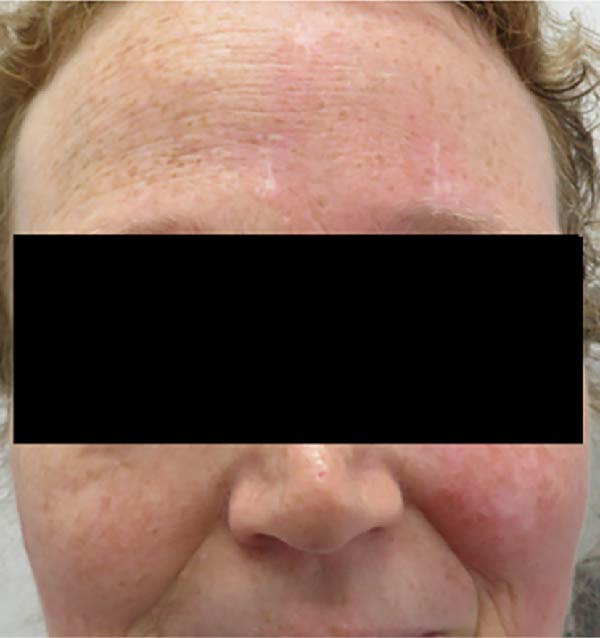
Photograph of patient taken following exertional exercise. Absence of expected erythema is evident across the right side of the face. Consent for publication of clinical photograph provided from patient following our institutional protocols.

The initial post‐operative course was uncomplicated. Six weeks post‐operatively, the patient noted asymmetry of facial erythema during strenuous exercise; noting erythema of only the left hemiface on exertion. On examination following an exercise challenge, a lack of expected erythema alongside anhidrosis was noted on the right side (Figure 3). It was confirmed with the patient that there was no pre‐existing issue with asymmetry of facial flushing prior to the thyroid surgery. There was no erythema of the face at rest and Pemberton’s test was negative. The eye examination was limited by the pre‐existing ptosis and visual impairment.

Subsequent neurological evaluation confirmed a right‐sided partial Horner syndrome, specifically involving the cutaneous sympathetic pathways. A limitation of this case report is that pharmacological confirmation with apraclonidine or cocaine was not performed; therefore, the diagnosis remains a clinical one. Following a multidisciplinary board recommendation, a completion thyroidectomy was performed to enable subsequent radioactive iodine treatment. Patient had an uneventful recovery from completion thyroidectomy. By 12 months post‐operatively, the patient noted the facial symptoms had improved, with anhidrosis only affecting the right forehead.

## 3. Discussion

Horner’s syndrome is an uncommon complication of thyroid surgery and is most frequently reported in association with lateral neck dissection. In contrast, its occurrence following thyroidectomy without lateral neck dissection is distinctly rare. A narrative review of the literature identified 21 reported cases of complete or partial Horner’s syndrome in adult patients following open thyroid surgery with or without central neck dissection, but without lateral neck dissection, across 17 publications (Table 1). The exclusion of paediatric cases, minimally invasive or endoscopic approaches, radical resections and lateral neck dissections underscores the rarity of this complication in conventional thyroidectomy. Importantly, a supplemental search using ‘Harlequin syndrome’, ‘thyroidectomy’, and ‘flushing asymmetry’ did not identify any additional cases, highlighting the exceptional nature of the exertional facial flushing asymmetry observed in our patient.

**Table 1:** Summary statistics from literature review.

Variable	Number of patients in category (denominator: *N* = 21)
Gender	
M	2
F	19
Operation	
Total thyroidectomy	10
Subtotal thyroidectomy	1
Selective thyroidectomy	1
Partial thyroidectomy	2
Right hemithyroidectomy	2
Left hemithyroidectomy	3
Unknown	2
Indication	
Goitre	10
Thyroid cancer	2
Nodule	5
Graves disease	1
Unknown	3
Side of symptoms	
Right	8
Left	11
Unknown	2
Presenting symptoms	
Ptosis only	2
Ptosis, miosis	5
Ptosis, enophthalmos	1
Ptosis, miosis, enophthalmos	3
Ptosis, miosis, anhidrosis	3
Ptosis, miosis, enophthalmos, anhidrosis	1
Ptosis, miosis, loss of ciliospinal reflex	1
Ptosis, miosis, heterochromia	1
Not specified	4
Management	
Oral steroid	1
Conservative	6
Not reported	14
Outcome	
Complete recovery	5
Partial recovery	8
No recovery	6
Not reported	2

Among these 21 cases, only three demonstrated the classical triad of ptosis, miosis and anhidrosis (Table 2), indicating that partial or incomplete Horner’s syndrome is more typical in this setting. No prior reports described exertional facial flushing asymmetry (Harlequin sign) following thyroidectomy, even in series involving lateral neck dissection. While congenital Horner’s syndrome with absent ipsilateral flushing has been described [4], this manifestation has not previously been associated with thyroid surgery. Disruption of sympathetic innervation to the skin is thought to impair thermoregulatory vasodilation and sweating on the affected side, sometimes resulting in compensatory erythema and hyperhidrosis contralaterally [4, 5, 26], a mechanism that plausibly explains our patient’s presentation.

**Table 2:** Cases of thyroidectomy +/− central neck dissection complicated by post‐operative Horner syndrome.

Author	Age, gender	Operation	Indication	Symptoms	Side of symptoms	Date of onset	Proposed mechanism	Management	Resolved Y/N (duration to resolution)
Arishi et al. [10]	29 F	Total thyroidectomy + central neck dissection	Papillary thyroid carcinoma	Ptosis, miosis and anhidrosis	Right	Immediately post‐op	Traction injury	Not reported	No
Aslankurt et al. [11]	42 F	Subtotal thyroidectomy	Multinodular goitre	Ptosis, miosis, anhidrosis	Right	Immediately post‐operative	—	Not reported	No
Cozzaglio et al. [8]	35 F	Total thyroidectomy	Graves disease	Ptosis, miosis, enophthalmos	Left	Post‐operative day two	Traction injury	Conservative	Yes (3 days)
de Silva et al. [12]	35 F	Hemithyroidectomy (left)	Thyroid nodule	Ptosis, miosis	Left	POD7	Damage to sympathetic chain due to aberrant anatomy	Not reported	Partial improvement at 3 months
Giannaccare et al.[13]	29 M	Total thyroidectomy	Thyroid nodule	Ptosis, miosis	Right	Immediately post‐op	Not reported	Not reported	No
Gillani and Jamil [14]	37 F	Unknown	Unknown	Ptosis, miosis	Right	Not reported	Not reported	Not reported	Not reported
Italiano et al. [15]	56 M	Total thyroidectomy	Multinodular goitre	Ptosis, miosis, enophthalmos	Right	Immediate post‐operative	Damage to recurrent laryngeal nerve in the presence of communicating fibres to sympathetic chain	Not reported	Slight improvement at 2 months
Janjua et al. [16]	27 F	Total thyroidectomy	Benign goitre	Ptosis, miosis enophthalmos and anhidrosis	Left	POD2	Traction injury	2 weeks prednisolone PO	Yes (6 months)
Neki et al. [17]	50 F	Hemithyroidectomy (right)	Thyroid nodule	Ptosis, miosis, anhidrosis and loss of ciliospinal reflex	Right	POD11	Ischaemic injury	Conservative	No
Ocak et al. [18]	29 F	Total thyroidectomy + bilateral central neck dissection	Medullary thyroid cancer (MEN2A)	Ptosis	Unknown	Immediately post‐operative	Traction injury	Conservative	Yes (2 months)
Perréard et al. [19]	46 F	Hemithyroidectomy (left)	Thyroid nodule	Ptosis, miosis	Left	Immediately post‐operatively	Not reported	Not reported	No
Rauniyar [20]	30 F	Hemithyroidectomy (right)	Thyroid nodule (follicular adenoma)	Ptosis	Right	Post‐operative day one	Traction injury	Conservative	Yes (8 months)
Reeve et al. [21]	Unknown F	Unknown	Unknown	Horners NOS	Not reported	Not reported	Not reported	Not reported	Yes (timeline unknown)
Seneviratne et al. [22]	36 F	Total thyroidectomy	Multinodular goitre	Ptosis, miosis, enophthalmos	Left	1 week post‐operative	Ischaemic injury +/− traction injury	Conservative	Incomplete recovery at 12 months
Smith and Murley [23]	50 F	Selective thyroidectomy	Bilateral nodular goitre with retrosternal progression	Ptosis, miosis	Right	POD2	Damage to sympathetic chain due to aberrant anatomy	Not reported	Partial improvement at 14 months
	51 F	Partial thyroidectomy	Diffuse toxic goitre	Horners NOS	Left	POD2	Damage to sympathetic chain due to aberrant anatomy	Not reported	Partial improvement at 3 years
	32 F	Partial thyroidectomy	Diffuse toxic goitre	Horners NOS	Left	POD1	Damage to sympathetic chain due to aberrant anatomy	Not reported	Significant improvement 8 months
	37 F	Thyroidectomy	Non‐toxic goitre	Ptosis, enophthalmos	Left	Unknown	Not reported	Not reported	Only partial improvement 12 years
	55 F	Thyroidectomy	Recurrent goitre	Horners NOS	Left	Immediate post‐operative	Possible ischaemic injury	Not reported	No
Solomon et al. [24]	65 F	Hemithyroidectomy (left)	Multinodular goitre	Ptosis, miosis, “questionable anhidrosis”	Left	Post‐operative day two	Ischaemic injury	Conservative	Partial improvement at 15 months
Ulusoy et al. [25]	23 F	Total thyroidectomy	Unknown	Ptosis, miosis heterochromia	Left	~ 1–2 years post	Not reported	Not reported	Not reported

Clinical outcomes reported in the literature are heterogeneous. Two cases documented concurrent neural injury, including recurrent laryngeal nerve irritation from clip placement [10] and inferior laryngeal nerve injury [15], suggesting a shared vulnerability of adjacent neural structures in the operative field. Only one report described active intervention with a short course of oral prednisolone, leading to complete resolution by 6 months [16], while most cases were managed expectantly. Recovery varied widely, ranging from spontaneous resolution within days to persistent symptoms for up to 12 years (Table 2), supporting the view that neuropraxia is a common underlying mechanism rather than permanent nerve transection.

Large cohort studies further reinforce the rarity of this complication. An incidence of approximately 0.2% has been reported in series of 495 [8] and 2636 [9] thyroidectomies, with the latter identifying Horner’s syndrome exclusively in patients undergoing lateral neck dissection. A multicentre study of 14,934 thyroid operations documented only a single case [27]. Similarly, an Australian series of 2208 thyroid and parathyroid procedures reported six cases (0.27%), five following lateral neck dissection and one following minimally invasive parathyroidectomy, with none occurring after thyroidectomy with or without central neck dissection alone. Collectively, these data highlight the exceptional nature of Horner’s syndrome following central compartment surgery, as seen in our patient.

Several mechanisms have been proposed to explain Horner’s syndrome following thyroidectomy without lateral neck dissection. Solomon et al. [24] described four principal aetiologies: traction‐induced neuropraxia of the cervical sympathetic chain during lateral retraction, compression from post‐operative haematoma, ischaemia‐induced nerve injury and damage to communicating fibres between the cervical sympathetic chain and the recurrent laryngeal nerve during intraoperative nerve identification. Traction injury is most frequently implicated. Lateral retraction for exposure may compress the carotid sheath [23], placing the sympathetic chain—typically located posterior or postero‐medial to it—at risk. Experimental data demonstrate the sensitivity of this structure, with Smith and Murley [23] showing that application of only 15 g of force for 1 min to a rabbit sympathetic chain was sufficient to produce Horner’s syndrome. Clinically, traction has been implicated during superior pole dissection in narrow operative fields [28] and during inferior pole mobilisation to expose the inferior thyroid artery [1], with neuropraxia explaining delayed and often incomplete recovery.

Post‐operative haematoma has been proposed as another mechanism through mass effect and secondary ischaemia [24]; however, none of the reviewed cases described a significant haematoma, and no clinically meaningful compressive collection was identified in our patient, rendering this explanation unlikely. Ischaemia‐induced nerve injury related to ligation of the inferior thyroid artery is also plausible. Ligation may compromise perfusion to the sympathetic chain, and dissection in this region brings the surgeon into close proximity with it [1]. This risk has long been recognised; Smith and Murley [23], citing De Quervain, noted the balance between avoiding the recurrent laryngeal nerve and approaching the sympathetic chain during inferior thyroid artery ligation. Aberrant artery anatomy and difficult haemostasis were present in some historical cases [23], and cadaveric studies have confirmed the close anatomical relationship between the inferior thyroid artery and the cervical sympathetic chain [6]. Anatomical communications between the recurrent laryngeal nerve and the cervical sympathetic chain, present in approximately 1.5%–3% of individuals [1], may be susceptible to traction [29] or injury during repeated nerve identification, particularly in the era of routine intraoperative neuromonitoring.

In our case, the delayed onset, absence of permanent ocular involvement and gradual improvement favour a neuropraxic injury rather than transection or irreversible ischaemia. While traction injury cannot be excluded, involvement of sympathetic–recurrent laryngeal nerve communicating fibres is also plausible. A compressive haematoma was unlikely given the minimal post‐operative collection, and ischaemia‐induced injury was considered less probable due to meticulous vessel ligation close to the gland capsule. This case, therefore, extends existing literature by describing an exceptionally rare presentation—exertional facial flushing asymmetry—in partial Horner’s syndrome following thyroidectomy without lateral neck dissection and underscores the importance of awareness of this complication during surgery.

## 4. Conclusion

We describe a rare case of partial Horner’s syndrome following thyroidectomy without lateral neck dissection, uniquely presenting with asymmetrical facial flushing. This report highlights an uncommon complication and reinforces the need for awareness of potential sympathetic chain injury even during surgery.

## Author Contributions

Conceptualisation, collected consent, supervision: Senarath Edirimanne. Data collection, writing – original draft: Eve Hopping. Writing – review and editing: Eve Hopping and Femi E. Ayeni. Project administration: Femi E. Ayeni.

## Funding

No funding was received for conducting this study. Open access publishing facilitated by The University of Sydney, as part of the Wiley ‐ The University of Sydney agreement via the Council of Australasian University Librarians.

## Disclosure

All authors have read and approved the final version of the manuscript. Senarath Edirimanne had full access to all of the data in this study and takes complete responsibility for the integrity of the data and the accuracy of the data analysis.

## Ethics Statement

Formal review by Nepean Blue Mountain Local Health Districts Research Ethics Committee was not required and an application for ethical approval was waived. All the procedures performed in this study were in accordance with the ethical standards of 1964 Helsinki Declarations.

## Consent

The patient provided informed consent for the publication of this case report.

## Conflicts of Interest

The authors declare no conflicts of interest.

## Data Availability

The authors have nothing to report.

## References

[1] Kus L. H. and Freeman J. L. , Miccoli P. , Terris D. J. , Minuto M. N. , and Seybt M. W. , The Rare Ones: Horner’s Syndrome, Complications From Surgical Positioning and Post-Sternotomy Complications, Thyroid Surgery: Preventing and Managing Complications, 2012, Wiley & Sons, 237–248.

[2] Lee J. H. , Lee H. K. , Lee D. H. , Choi C. G. , Kim S. J. , and Suh D. C. , Neuroimaging Strategies for Three Types of Horner Syndrome With Emphasis on Anatomic Location, American Journal of Roentgenology. (2007) 188, no. 1, W74–W81, 10.2214/AJR.05.1588, 2-s2.0-34548801719. 17179330

[3] Lyons A. J. and Mills C. C. , Anatomical Variants of the Cervical Sympathetic Chain to Be Considered During Neck Dissection, British Journal of Oral and Maxillofacial Surgery. (1998) 36, no. 3, 180–182, 10.1016/S0266-4356(98)90493-4, 2-s2.0-0031805720. 9678881

[4] Saito H. , Congenital Horner’s Syndrome With Unilateral Facial Flushing, Journal of Neurology, Neurosurgery & Psychiatry. (1990) 53, no. 1, 85–86, 10.1136/jnnp.53.1.85, 2-s2.0-0025174629. 2303838 PMC1014106

[5] Bistline A. K. and Jones E. , Harlequin Syndrome: Discovery of an Ancient Schwannoma, Cutis. (2022) 109, no. 4, E18–E20, 10.12788/cutis.0514. 35659839

[6] Civelek E. , Karasu A. , and Cansever T. , et al.Surgical Anatomy of the Cervical Sympathetic Trunk During Anterolateral Approach to Cervical Spine, European Spine Journal. (2008) 17, no. 8, 991–995, 10.1007/s00586-008-0696-8, 2-s2.0-49249122414. 18548289 PMC2518767

[7] Ebraheim N. A. , Lu J. , Yang H. , Heck B. E. , and Yeasting R. A. , Vulnerability of the Sympathetic Trunk During the Anterior Approach to the Lower Cervical Spine, Spine. (2000) 25, no. 13, 1603–1606, 10.1097/00007632-200007010-00002, 2-s2.0-0033924646. 10870134

[8] Cozzaglio L. , Coladonato M. , and Doci R. , et al.Horner’s Syndrome as a Complication of Thyroidectomy: Report of a Case, Surgery Today. (2008) 38, no. 12, 1114–1116, 10.1007/s00595-007-3741-z, 2-s2.0-57049186468. 19039637

[9] Lee Y. S. , Nam K.-H. , Chung W. Y. , Chang H.-S. , and Park C. S. , Postoperative Complications of Thyroid Cancer in a Single Center Experience, Journal of Korean Medical Science. (2010) 25, no. 4, 541–545, 10.3346/jkms.2010.25.4.541, 2-s2.0-77954010672. 20357995 PMC2844597

[10] Arishi A. A. , Abualhana F. , and Sferra J. , Horner’s Syndrome Following Thyroid Surgery, Cureus. (2023) 15, no. 9, 10.7759/cureus.45825. PMC10591229 37876407

[11] Aslankurt M. , Aslan L. , Çolak M. , and Aksoy A. , Horner’s Syndrome Following a Subtotal Thyroidectomy for a Benign Nodular Goitre, BMJ Case Reports. (2013) 2013, 10.1136/bcr-2013-009907, 2-s2.0-84880064203, bcr2013009907. PMC3702989 23771972

[12] de Silva W. D. , de Soysa M. S. , and Perera B. L. , Iatrogenic Horner’s Syndrome: A Rare Complication of Thyroid Surgery, Ceylon Medical Journal. (2010) 55, no. 4, 10.4038/cmj.v55i4.2639, 2-s2.0-79952639567, 136. 21341635

[13] Giannaccare G. , Gizzi C. , and Fresina M. , Horner Syndrome Following Thyroid Surgery: The Clinical and Pharmacological Presentations, Journal of Ophthalmic and Vision Research. (2016) 11, no. 4, 442–444, 10.4103/2008-322X.194146, 2-s2.0-84999273596. 27994816 PMC5139559

[14] Gillani S. S. and Jamil A. Z. , Case Report on Horners Syndrome Following Thyroidectomy, Pakistan Journal of Ophthalmology. (2019) 35, no. 1.

[15] Italiano D. , Cammaroto S. , Cedro C. , Bramanti P. , and Ferlazzo E. , Neurological Sciences, 2011, 32, no. 3, 10.1007/s10072-010-0451-x, 2-s2.0-79960116580, 531. 21088976

[16] Janjua M. H. , Iftikhar S. , Sarwar M. Z. , Farooq M. S. , and Naqi S. A. , Horner Syndrome—A Rare Complication After Thyroidectomy for Benign Thyroid Swelling, Nigerian Journal of Clinical Practice. (2021) 24, no. 12, 1852–1854, 10.4103/njcp.njcp_419_20. 34889796

[17] Neki N. S. , Shergill G. S. , Singh A. , Kaur A. , Nizami S. , and Sidhu P. B. , Post Thyroidectomy Horner’s Syndrome—Expect the Unexpected!, Bangladesh Journal of Medical Science. (2017) 16, no. 4, 600–601, 10.3329/bjms.v16i4.33620, 2-s2.0-85028349009.

[18] Ocak S. , Alakus H. , Duymus M. E. , Kaya M. , Karadayi K. , and Colak E. , Horner’s Syndrome: A Rare Complication of Thyroid Cancer Surgery, Translational Surgery. (2016) 1, no. 3, 10.4103/2468-5585.191505, 88.

[19] Perréard M. , Bailleul H. , and Babin E. , Post-thyroidectomy Horner’s syndrome, European Annals of Otorhinolaryngology, Head and Neck Diseases. (2019) 136, no. 5, 419–420, 10.1016/j.anorl.2019.05.013, 2-s2.0-85072586224. 31253593

[20] Rauniyar N. , Horner’s Syndrome in Thyroidectomy Patient: Case Report, SAGE Open Medical Case Reports. (2023) 11, 10.1177/2050313X231220811, 2050313X231220811. PMC10752067 38152685

[21] Reeve T. S. , Coupland G. A. E. , Johnson D. C. , and Buddee F. W. , The Recurrent and External Laryngeal Nerves in Thyroidectomy, Medical Journal of Australia. (1969) 1, no. 8, 380–382, 10.5694/j.1326-5377.1969.tb92166.x. 5782083

[22] Seneviratne S. A. , Kumara D. S. , and Drahaman A. M. P. , Horner’s Syndrome: An Unusual Complication of Thyroidectomy: A Case Report, Journal of Medical Case Reports. (2016) 10, no. 1, 10.1186/s13256-016-1072-7, 2-s2.0-84995428273, 300. 27784321 PMC5081681

[23] Smith I. and Murley R. S. , Damage to the Cervical Sympathetic System During Operations on the Thyroid Gland, Journal of British Surgery. (1965) 52, no. 9, 673–675, 10.1002/bjs.1800520909, 2-s2.0-0007509663. 14338315

[24] Solomon P. , Irish J. , and Gullane P. , Horner’s Syndrome Following a Thyroidectomy, Journal of Otolaryngology. (1993) 22, no. 6, 454–456. 8158744

[25] Ulusoy M. O. , Kıvanç S. A. , Atakan M. , and Mayalı H. , Post-Thyroidectomy Iatrogenic Horner’s Syndrome With Heterochromia, Journal of Current Ophthalmology. (2016) 28, no. 1, 46–47, 10.1016/j.joco.2016.02.004, 2-s2.0-84979725677. 27239603 PMC4881223

[26] Barbagli G. , Soto-Rubio D. , and Pacheco-Barrios N. , et al.Harlequin Sign Due to an Upper Thoracic Paravertebral Lesion. A Systematic Review of the Literature, Journal of Clinical Neuroscience. (2024) 129, 10.1016/j.jocn.2024.110850, 110850. 39342897

[27] Rosato L. , Avenia N. , and Bernante P. , et al.Complications of Thyroid Surgery: Analysis of a Multicentric Study on 14,934 Patients Operated on in Italy Over 5 Years, World Journal of Surgery. (2004) 28, no. 3, 271–276, 10.1007/s00268-003-6903-1, 2-s2.0-1642408767. 14961204

[28] Chen H. , Sun Y. , Tang M. , and Zhang F. , Horner Syndrome Immediately After Deep Dissection of Upper Thyroid Pole: A Case Report and Review of the Literature, Innovative Surgical Sciences. (2024) 9, no. 1, 63–66, 10.1515/iss-2023-0056. 38826629 PMC11138407

[29] Harding J. L. , Sywak M. S. , Sidhu S. , and Delbridge L. W. , Horner’s Syndrome in Association With Thyroid and Parathyroid Disease, ANZ Journal of Surgery. (2004) 74, no. 6, 442–445, 10.1111/j.1445-1433.2004.03030.x, 2-s2.0-3042760043. 15191478

